# Ozone Layer Depletion and Emerging Public Health Concerns - An Update on Epidemiological Perspective of the Ambivalent Effects of Ultraviolet Radiation Exposure

**DOI:** 10.3389/fonc.2022.866733

**Published:** 2022-03-10

**Authors:** Sheikh Ahmad Umar, Sheikh Abdullah Tasduq

**Affiliations:** ^1^ Department of Biological Sciences, Academy of Scientific and Innovative Research (AcSIR), Ghaziabad, India; ^2^ Pharmacokinetics-Pharmacodynamics (PK-PD) and Toxicology Division, Council of Scientific and Industrial Research-Indian (CSIR) Institute of Integrative Medicine, Jammu, India

**Keywords:** ultraviolet radiation (UV), skin photodamage, ozone layer depletion, vitamin D deficiency, sun protection measures, food fortification

## Abstract

Solar ultraviolet (UV) radiation exposure is the primary etiological agent responsible for developing cutaneous malignancies. Avoiding excessive radiation exposure, especially by high-risk groups, is recommended to prevent UV-induced photo-pathologies. However, optimal sun exposure is essential for the healthy synthesis of about 90% of vitamin D levels in the body. Insufficient exposure to UV-B is linked to vitamin D deficiency in humans. Therefore, optimal sun exposure is necessary for maintaining a normal state of homeostasis in the skin. Humans worldwide face a major existential threat because of climate change which has already shown its effects in several ways. Over the last 4 to 5 decades, increased incidences in skin cancer cases have led international health organizations to develop strong sun protection measures. However, at the same time, a growing concern about vitamin D deficiency is creating a kind of exposure dilemma. Current knowledge of UV exposure to skin outweighs the adverse effects than the beneficial roles it offers to the body, necessitating a correct public health recommendation on optimal sun exposure. Following an appropriate recommendation on optimal sun exposure will lead to positive outcomes in protecting humans against the adverse effects of strict recommendations on sun protection measures. In this short review, we spotlight the ambivalent health effects of UV exposure and how ozone layer depletion has influenced these effects of UVR. Further, our aim remains to explore how to lead towards a balanced recommendation on sun protection measures to prevent the spurt of diseases due to inadequate exposure to UV-B.

## Introduction

Ultraviolet radiation, the main component of sunlight, is divided into three categories, UV-A, UV-B and UV-C based on the wavelength and energy status (1, 2). UV-B has high energy and potential than UV-A to cause the biological damage (3). In contrast, UV-C is retained by the ozone layer and never reaches the lower atmosphere (4). The average UV dose across the globe varies with geographical location and on daily to seasonal timescales. The total ozone is generally lowest at the equator and highest in mid-latitude and Polar regions. This way, the global distributional pattern of UV index varies with the latitude, altitude, cloud cover and haze and is further complicated amid the ozone layer depletion scenario. Therefore, no definite UV dose can be attributed to a particular region across the globe (3). However, substantial UV index changes have happened over the last few decades due to ozone layer depletion that has significantly increased the global burden of skin cancer incidences. The recovery of the ozone layer will depend on how countries abide by the Montreal Protocol treaty terms by the participating countries in times to come and if they take the treaty terms very seriously (5). The impact of future climate change on the ozone layer will vary between the tropics, mid-latitudes and Polar regions and strongly depends on future emissions of ozone-depleting substances. During the long recovery period, volcanic eruptions could temporarily reduce the global ozone levels for several years. Together, these all things will be playing pivotal roles in controlling the global UV changes and the after-effects that ozone layer depletion can have on the different life forms across the globe (6–10).

Likely, fair-skinned individuals are at the highest risk of developing the UV mediated photodamage responses differentially varying with different skin types (11). Yet, most skin types are prone to sun damage with ever-increasing exposure to UV radiation (12). UV radiation not only affects humans but the animal, plant and marine life is also significantly impacted (13). There is a growing concern that ozone layer depletion may lead to the loss of many threatened plant species and disrupt the global food security (14). However, plants have built the ability to respond and adapt to high UV levels; they can be affected directly due to high UV radiations (15), affecting most plant species’ survival (16). The adverse effects of ozone depletion on marine ecosystems can be many, including reducing the population of tiny marine organisms due to small increases in UV, significantly disrupting the marine ecosystem (17). An increase in UV-B radiations reaching the earth’s surface may also disrupt and change the natural pattern of biogeochemical cycles and contribute to biosphere-atmosphere feedback, which could have even more deleterious effects on different life forms (18). Although the risks of UV radiation overexposure are known and many **(** **Table 1** **),** the general public have been made to think about the ill effects of UV and not to weigh the merits of UV radiation exposure being essentially crucial for Vitamin D synthesis. The production of 10μg (400 IU) of vitamin D per day takes approximately 1/3 of the time needed to reach the Minimal Erythemal Dose (MED) for an effective skin area of 600cm^2^ for skin phototype III. It indicates that UV exposure has strict bodily requirements to synthesize these required amounts of Vitamin D for proper bone formation and function (19). The optimal UV dose for vitamin D production varies significantly depending on the physiological and pathological condition of every individual. The best assessment of these personal attributes can allow people to find their unique “Goldilock” zones of exposure time. This mini-review highlights the ambivalent biological effects of UVR and how these effects can further modulate if the overhead ozone cover continues to change negatively in the future. It further highlights how to lead towards a suitable public health recommendation on optimal sun exposure amid the climate change triggered ozone layer depletion.

**Table 1:** Classification of skin types/skin color types and burns/tans in the skin after sun exposure.

S.No	Exposure category	UVI range	Skin Type classification	Burns/Tans after sun exposure	Diseases due to inadequate UVR exposure/vitamin D levels in body	Diseases due to excessive UVR exposure
01	Low	<2	VI • Naturally black skin • High Eumelanin and low Phaeomelanin • Melano- protected	• No • No	**Cancers:** • Prostate • Breast cancer • Colorectal cancer • Ovary cancer • Non-Hodgkin lymphoma **Autoimmune diseases:** • Multiple sclerosis • Type 1 diabetes • Rheumatoid arthritis **Psychiatric disorders:** • Seasonal affective disorder • Mood disorders • Schizophrenia • **Insufficient vitamin D levels** • Rickets • Osteomalacia • Osteoporosis	**Effects on the skin** **Acute** • Sunburn • Photodermatoses **Chronic** • Cutaneous malignant melanoma • Cancer of the lip • Basal cell carcinoma • Squamous cell carcinoma • Chronic sun damage/solar keratoses **Effects on the Eyes** **Acute** • Acute photokeratitis and conjunctivitis • Acute solar retinopathy **Chronic** • Climatic droplet keratopathy • Pterygium • Pinguecula • Squamous cell carcinoma of the cornea • conjunctiva • Cataract • Ocular melanoma • Macular degeneration **Other effects** • Suppression of cell-mediated immunity • Increased susceptibility to infection • Impairment of prophylactic immunization **Indirect effects** • Effect on climate, food supply, disease vectors, atmospheric chemistry
02	Moderate	2-5	V • Naturally brown skin • High Eumelanin and low Phaeomelanin) • Melano- protected	• Negligible • Negligible		
03	High	6-7	IV • Light skin • High phaeomelanin and low eumelanin • Melano- competent	• Sometimes seldom • Always usually		
04	Very High	8-10	III • Light skin and Freckles • High phaeomelanin and low eumelanin) • Melano- competent	• Sometimes seldom • Always usually		
05	Extreme	11+	II I • Pale • None or very little eumelanin or phaeomelanin (albinism) • Melano-compromised	• Always usually • Sometimes seldom		

UV radiation exposure categories, UV index range and diseases due to inadequate/excessive exposure to UVR.

## Health Promoting Effects Of Ultraviolet Radiation Exposure To Skin

Despite the numerous health concerns that UV radiation exposure comes with. It has several health promoting advantages that make sun exposure a kind of necessary evil having ambivalent effects to human body. Some of the prominent health promoting effects of UV exposure is summarized below.

### UV Induced Melanogenesis Acts as a Natural Sunscreen 

Melanin is a coloring pigment that is synthesized by the melanocytes in skin and its synthesis is promoted during sunlight exposure due to its UV portion. This is the same pigment that is responsible for giving colour to skin. It is also involved in primary natural defenses against UV-induced DNA damage (20). Special cells synthesize melanosomes under the skin, which produce, store and transport melanin and are absorbed by skin keratinocytes forming a protective, UV-blocking shell around the cells’ nuclei. In the cytosol of keratinocytes, melanosomes form a critical shield of DNA by forming perinuclear caps exhibiting photoprotection (21). These responses against UV depend on different parameters including the production, distribution, quantity and type of melanin synthesized in melanocytes and the content transferred to keratinocytes (22). The photoprotective effect of melanin is achieved in part by acting as a physical barrier and as an absorbent filter that reduces the penetration of UV through the epidermis (23). Some persons however, suffering from diseases albinism and vitiligo have a faulty melanin production and are highly susceptible to the effects of UV exposure (24). Epidemiological data strongly suggest and support the protective role of melanin in skin against the UV exposure induced cellular damage as there exists an inverse correlation between skin pigmentation and the incidence of sun-induced skin cancers. Research has suggested that subjects with White skin including albino’s, are more likely to develop skin cancer by about 70 times than subjects with Black skin (25). Other important properties of melanin, especially eumelanin are its functions acting as a free radical scavenger and superoxide dismutase that reduce oxidative stress in skin cells (26).

### UV Phototherapy in Treatment of Numerous Cutaneous Disorders

UV light-based phototherapy is the most frequently used method and has a long, successful history in the management of numerous cutaneous disorders. UV-based phototherapy works by regulating the inflammatory component and inducing apoptosis of pathogenic cells, quickly transforming the microenvironment of UV-exposed skin (27). Phototherapy effects include proapoptotic, anti-fibrotic, pro-pigmentary, immunomodulatory, anti-pruritic and pro-prebiotic that promotes clinical prognosis and outcomes in various skin diseases such as psoriasis, atopic dermatitis, vitiligo, scleroderma, and cutaneous T-cell lymphoma (CTCL) (28). This therapy works by reacting with many elements essentially, chromospheres, metabolic byproducts, innate immune receptors, neurotransmitters and mediators such as chemokines, antimicrobial peptides, and platelet activating factor (PAF) that simultaneously shape the immunomodulatory effects of UV and their interplay with the microbiota of the skin. Most of the positive effects of solar radiation are mediated *via* ultraviolet-B (UVB) induced production of vitamin D in skin.

#### Psoriasis

UVB phototherapy is used in the treatment of psoriasis, which is an inflammatory skin disease, characterized by keratinocyte hyper proliferation. Even though UVB phototherapy is a standard treatment for psoriasis, however, the underlying molecular mechanisms of its efficacy are not completely understood. It is speculated that the therapeutic effectiveness of phototherapy is mainly due to its antiproliferative properties (29).

#### Vitiligo

Vitiligo is a de-pigmentation skin disorder and appears to be a combination of genetic effects in both the immune system and in the melanocytes, resulting in melanocyte destruction (30). Multiple treatments are recommended in the treatment of vitiligo, especially phototherapy with narrowband UVB radiation and excimer laser (308 nm) with/without the topical application of calcineurin antagonists (31). Role of phototherapy in treating vitiligo is supported by the fact that sun-exposed lesions tend to show follicular repigmentation during the summer months in many patients and the effect is transient but repeatable (32). The protective effect of phototherapy in patients with vitiligo is not completely elucidated. It is thought that re-pigmentation after phototherapy may be due to activation, proliferation, and migration of these affected melanocytes to the epidermis, where they form perifollicular pigmentation islands. Furthermore, UV light works as an immunosuppressant in skin that may also be playing a role in initiating re-pigmentation in melanocytes (33).

#### Atopic Dermatitis

UV light-based phototherapy is also used in the treatment of Atopic dermatitis which is a chronic inflammatory skin disease. Narrowband UVB und UVA-1 is the frequently used treatment setting in atopic dermatitis and in other T cell mediated inflammatory skin diseases. UV light exposure has direct phototoxic effects on T-lymphocytes causing gradual reduction of the inflammatory infiltrate and a concomitant improvement of patients’ skin affected with Atopic dermatitis (34).

#### Multiple Sclerosis

UV-B phototherapy is also used in the treatment of Multiple Sclerosis and has been found to prevent multiple sclerosis like symptoms in a mouse model regardless of the presence of vitamin D or the vitamin D receptor (35). People who are exposed to medium-to-high levels of ultraviolet-B radiation have a lower risk of developing Multiple Sclerosis. Given that UV-B exposure triggers the synthesis of vitamin D in the skin, many researchers have linked MS to a lack of vitamin D due to low sunlight exposure. However, researchers using a mouse model for MS have showed that exposure to UV-B prevent MS-like symptoms without increasing the vitamin D levels challenging a direct link between vitamin D and MS (36).

### UV Exposure Induced Nitric Oxide (NO) Reduces Blood Pressure and Mitigates Cardiovascular Disorders

Nitric oxide (NO) is a gaseous lipophilic free radical cellular messenger and plays an important role in the protection against cardiovascular diseases. Research has suggested that reduced bioavailability of NO is one of the central and critical factors common to cardiovascular diseases, although it is unclear whether this is a cause or results due to endothelial dysfunction (37). Low concentrations of NO^•^ has been found to protect cultured keratinocytes from oxidative stress and apoptosis. However, the underlying mechanisms are still unknown. A study demonstrated that UVA –irradiation to healthy individuals lead to a sustained reduction in blood pressure and these effects may be mediated by mechanisms that are independent of vitamin D and exposure to UV alone, but through UVA-induced NO^•^ and nitrite (32). NO_2_^-^ is not only known dilating the blood vessels, but also protect organs against ischemia/reperfusion damage and can be externally delivered to the systemic circulation to exert coronary vasodilator, cardio protective as well as antihypertensive effects. It is also proposed that UVA-induced NO^•^ have antimicrobial effects and is involved in cutaneous wound healing and has antitumor activity as well (38). However, despite its numerous health benefits, NO^•^ has with it toxic effects and that is why it is also known as a Janus molecule. UV exposure-produced NO^•^ can promote many local and systemic UV-induced responses including erythema, edema, inflammation, premature aging and immunosuppression. However, its role in the development and progression of skin cancer remains unclear.

### UV Exposure Improves Mood

Sun exposure in non-erythemic doses is considered as a pleasant one. Exposure to sunlight has been linked to improved energy and elevates the mood (39). People feeling better and relaxed after tanning partly support this phenomenon. UV radiation leads to production of an opioid β-endorphin *via* stimulation of the POMC promoter (Pro-opiomelanocortin) in keratinocytes and when released into the bloodstream may reach the brain in sufficient concentrations to elevate mood (32). However, only few studies have demonstrated the mood improving roles of increased β-endorphin levels in blood in healthy volunteers. Furthermore, both sunlight and darkness are involved in triggering the release of hormones in brain. Exposure to sunlight increases the release of serotonin from the brain, associated with boosting mood and helps a person feel calm. However, at night, dark light triggers the synthesis of melatonin in brain, responsible for sleep. Inadequate exposure to sun light can cause dip in the levels of serotonin associated with a risk of major depression with seasonal pattern (formerly seasonal affective disorder). This is a form of depression triggered by the changing seasons (40). Additionally, sunbathing or tanning beds have a potential to reduce pain in patients with fibromyalgia. A study has reported that patients experienced a greater short-term decrease in chronic pain after exposure to UV compared with non-UV radiation exposure (32).

## Impact Of Ozone Layer Depletion On The Effects Of Uv Exposure

### Disruption in the Evolutionarily Mediated Adaptation of Life Forms to Atmospheric UVR Changes

Living organisms have significantly evolved with time as the atmosphere they habituate changes continuously. The development of skin pigmentation responses in humans are likely due to selection pressures related to ambient ultraviolet radiation exposure. It has significantly influenced the migration of people from areas of high UVR index to regions of low UVR (41). Variation in the global distributional pattern of ultraviolet radiation poses diverse and differential effects based on latitude and altitude. There is always a debate going on among the researchers weighing out the adverse effects of UV exposure, despite the several benefits to different life forms, including humans, animals and vegetation. Ultraviolet radiation exposure requirements promoting healthy vitamin D synthesis in skin meant that people developed darker skin pigmentation at places of low latitude with high ambient UVR intensity, offering them protection from the effects of UVR. In contrast, those at higher latitudes have fairer skin as an evolutionarily developed trait to potentiate the vitamin D production from low ambient exposure (42). This contrasting requirement of latitudinal orientation set by humans throughout evolution for the healthy synthesis of vitamin D levels have changed as people adopted multiple sun protection measures and avoided sunlight exposure in pursuit of escaping the sun damage. These factors challenged the natural setup system and have adversely manifested into the development of various related skin and skeleton pathologies that requires regular ambient doses of radiation to function normally (43). In the last several decades, intensive human migration has interfered with the natural skin pigmentation patterns suited to the environment humans are born, grow, and evolve. The migration of people who are dark-skinned to areas of high latitude increases their chances of developing rickets and osteomalacia later in their life due to unhealthy/sub-optimal vitamin D synthesis (44). Fair-skinned populations at the other end are experiencing a steep rise in the number of melanoma and non-melanoma skin cancers migrating to low latitude areas from their natural habitats. Additionally, lifestyle changes combined with behavioural and cultural changes meant that humans were exposed to UV radiation than ever before, further compounding the problems the body responds to these changes (45). Increased incidences in skin cancer-related cases, cataracts, particularly in high-risk cataract belts of the world, improper vitamin D levels, skeletal and other cardiovascular diseases in the last 4-5 decades have gained considerable attention of the world scientific community on how to curb this sharp rise associated with the inappropriate exposure to sunlight. It has also led to a search for a viable solution based on one health approach, in addition to strict sun protection measures and vitamin D complementation from external sources (46).

## Ozone Layer Depletion And Global Burden Of Increase In The Uv Exposure Mediated Disease Incidences

**Figure 1:**
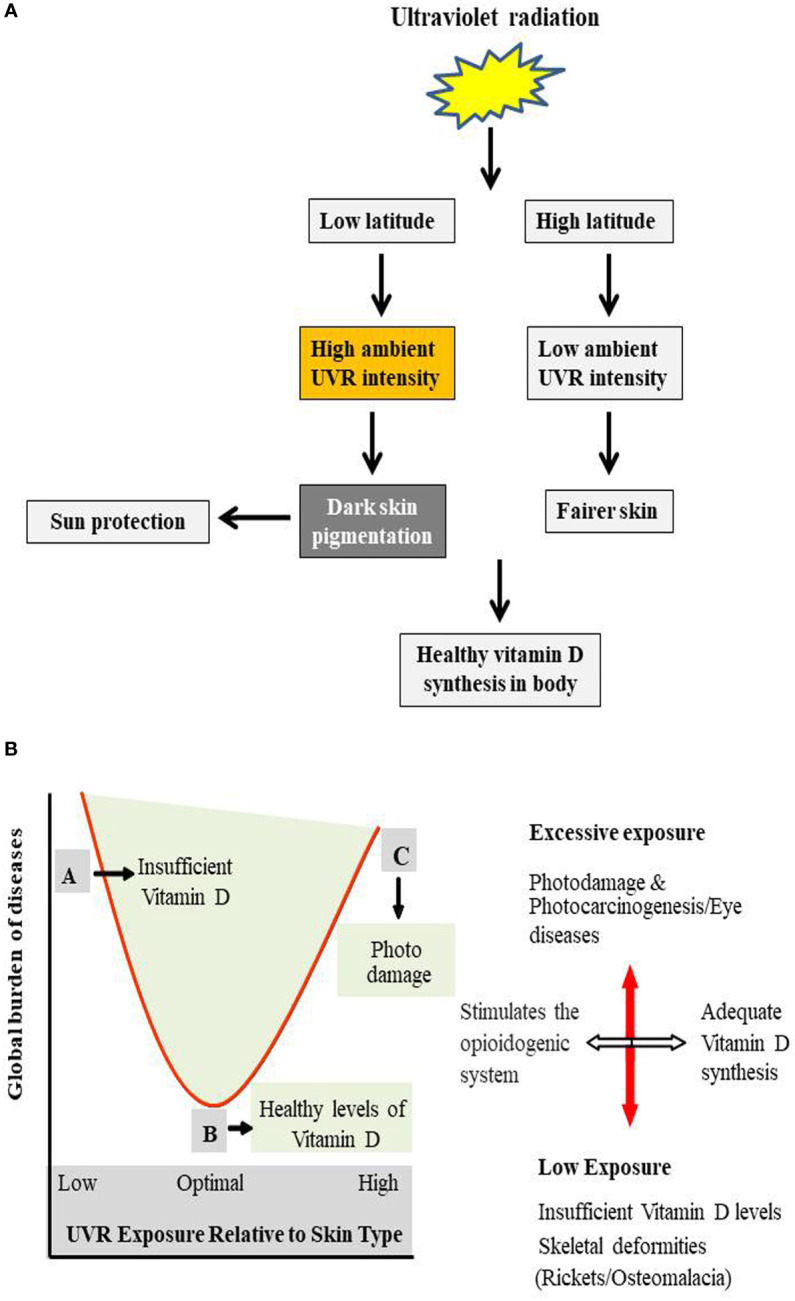
**(A)** Evolutionarily mediated adaptation of life forms to differential atmospheric UVR levels. UV radiation exposure requirements promoting healthy vitamin D synthesis in skin meant that people developed darker skin pigmentation at places of low latitude with high ambient UVR intensity, offering them protection from the effects of UVR. While, those at higher latitude have fairer skin as an evolutionarily developed trait to potentiate the insufficient vitamin D production from low ambient exposure of UV to skin. **(B)** Ambivalent effects of UVR exposure to skin. Schematic diagram showing relationship between benefits of optimum UVR exposure, ill effects of inadequate exposure and the global burden of diseases due to inappropriate UV exposure. A represents insufficient UVR exposure responsible for the improper vitamin D levels in the body leading to skeletal abnormalities and other indirect effects of low ambient UV exposure. B represents optimal UVR exposure required for the essential and healthy synthesis of vitamin D in the body and also stimulates the opioidogenic system in the brain. C shows high UVR exposure leading to skin and ocular malignancies especially, in fair-skinned individuals. Both A & C are related to inappropriate exposure of UVR to the skin.

The ozone layer acts as a natural filter, absorbing most of the sun’s ultraviolet rays coming towards the earth’s atmosphere. Changes to the ozone layer starting in the latter part of the 20^th^ century led to an increase in the proportion of UV-B radiations reaching the earth’s surface. It potentially disrupts the biological life and processes, including damaging several non-life entities, including polymer-based materials, such as thermoplastics, thermosets and composites used as replacements for traditional building materials, through a phenomenon known as chalking (10, 47). Ozone layer depletion resulted from rapid industrialization, high consumption of chlorofluorocarbons (CFCs) and halons, and global warming have further worsened the problem towards more destruction (48). This loss of ozone is associated with increased levels of radiation reaching the earth’s surface. Still, lower atmospheric pollution makes it difficult to assess changes in UVR patterns using ground-based measurements. Further, there are mainly three ways climate changes and their after-effects have shown their adverse effects on different life forms; stratospheric ozone depletion, increase in surface temperature due to global warming, and air pollution. Research suggests that globally and especially among the fair-skinned populations, melanoma rates are increasing by 4% to 5% annually. Further, increased temperatures/heat also has an impact on carcinogenesis. Past research has shown that non-melanoma skin cancer risk increases for every one-degree rise of temperature, suggesting that as the planet continues to warm, there’s the possibility that rising temperatures could further drive and amplify the induction of skin cancer cases due to UV radiation over exposure (49). Although this temporal trend in the increased incidence of non-melanoma skin cancers is difficult to determine, the increase is not simply a result of increased epidemiological surveillance and detection. Specific studies carried out in Australia, Canada and the US indicate that between the 1960-1980s, the prevalence of non-melanoma skin cancers increased by a factor of more than two when examined concerning personal UV exposure. It indicates a positive correlation between climate change mediated high UV exposure to increased skin cancer cases. The increase in skin cancers is most frequent in some parts of the body commonly exposed to the sun, such as the face and hands, implying that long-term, repeated UV radiation exposure is a major causal factor. Also, there exist a clear relationship between increased incidence of non-melanoma skin cancers with decreasing latitude within some countries, i.e. where there are high UV radiation levels (50). It also suggests that this increase in skin cancer incidences is not simply a result of increased epidemiological surveillance and detection (51). Further, studies in the Antarctic have shown that UV-B can double during the yearly ozone hole process measured at the earth’s surface (52). Other research studies found that in areas with little or no atmospheric pollution, UVR levels reaching the earth can be even more than observed before the ozone layer depletion started. Similarly, lower ambient levels of UV radiation are detected in areas with intense atmospheric pollution, and highly dense smog remaining through most part of the year (53). Also, studies in experimental animals have shown that elevated temperatures enhance the UV-induced skin cancer compared to that at room temperature. In an intriguing analysis, assuming that ambient temperature would have a similar effect in humans, speculates that long-term elevation of temperature by two °C as a consequence of global warming coupled climate change would increase the carcinogenic effects of solar UV by 10% (54). Further, experimental mouse models have shown that the carcinogenic effects of UV radiation increase by 5% per °C rise in temperature (55). However, research is still going on, and there is no clear evidence of how the increase in surface temperature can increase the carcinogenic effects of UV radiation. Increased incidences in the diseases associated with insufficient vitamin D levels have also been noticed in the past many decades, probably due to avoiding UV-B exposure or using sun protection gears at occupational places despite the increase in UVR index. These ambivalent and emerging health effects of UVR on net loss or gain will thus depend on various parameters, including the migratory pattern of people influencing their exposure leading to an imbalance in optimal exposure to what is required naturally (56) **(** **Figure 1** **).** The social cost of these diseases due to indecent exposure to UVR and the financial burden it entails is overwhelming as human sufferings continue to increase. Further, ozone depletion has come with a dichotomous nature for humans to avoid sun exposure to prevent skin-related pathological conditions or keep taking sunbaths for healthy vitamin D synthesis (57). To further clarify this ambiguity, a comprehensive report by United Nations Environment Programme estimated an additional burden of 4500 melanoma cases and 300,000 non-melanoma cases if there is a 10% decrease in the ozone layer, and these figures are in addition to those cases that happen under normal circumstances. WHO has also made it clear that among the total cataract cases that occur annually the world over, an estimated 3 million cases per year, accounting for 20% of total cases, could be due to UV exposure. Also, for each 1% sustained decrease in ozone, a 0.5% increase in the number of cataracts could be due to UV exposure alone (9, 58–60). These statistics ask for a strategic global action plan as total avoidance of UV exposure is already ruled out due to other problems manifested in the absence of sun exposure. Further, human evolution at low latitudes where sunlight is more intense and their migration towards high latitude areas have been driven by competing for folate deficiency and vitamin D, both of which are phenomena driven by UV exposure (61). In addition to the concerns due to ozone layer depletion, anthropogenic impacts that are more intense than ever can magnify the effects of UV on both humans and the rest of the environment (62). The Montreal Protocol, though, resulted in a considerate reduction in the emissions of CFCs and halons responsible for damaging the ozone layer that has already started to replete itself. There is still an estimated additional burden of 33,000 melanoma/non-melanoma cancer cases attributed to ozone layer depletion (63, 64). Climate change due to global warming is another factor that could play a role in potentiating or magnifying the cancer-causing potential of UV. Although, significant improvements were made by countries in reducing the global consumption of ozone-depleting substance by some 98% under the Montreal Protocol treaty, the full recovery of ozone is not possible for some decades as the depleting substances continue to stay in the atmosphere for years together. Future outcomes will therefore depend on how countries abide by the treaty terms and conditions, thereby preventing the further loss (65).

## Vitamin D Deficiency And Optimization Of Appropriate Public Health Recommendation On Uvr Exposure

Although the ambivalent effects of UV radiation exposure are known, we are yet to understand which particular dose of UV is adequate and the one that is excessive to different life forms (66). This standardization of dose is further complicated amid the ozone layer depletion scenario combined with global warming that has significantly impacted the optimization of optimal, suboptimal and above optimal exposure levels of UVR and its post-effects (7). These factors necessitate a suitable public health policy in the wake of these distribution pattern changes of global UV exposure to life on earth. There are specific solutions to these problems, but the outcome is subjective. If we can maintain sufficient vitamin D levels from outside sources, UVR exposure can be optimized, and excessive exposure can be avoided accordingly. Before doing that, we have to rule out any other important function of UV exposure-related biological role in humans and requires further research to deconstruct the hidden facts (67, 68). For this, research has to define what vitamin D sufficiency to the body means and how much vitamin we have to take from the outside sources. This requirement will further depend on age, type of skin, area of location and typical dietary patterns. Only then a counterfactual exposure be defined, which can be a kind of minimum theoretical risk. This way, we can determine the limit of radiation exposure without significantly altering or impacting the vitamin D status in the body (69–71).

Further new structural model-based studies can be used to calculate the burden of diseases due to excessive UVR exposure. Inclusions on human skin pigmentation, physical inactivity, diet patterns, quality of healthcare system, behavioural sun exposure and latitude need to be considered to figure out a near accurate correlation of UVR exposure and its diverse effects. Earlier assessments on quantification of the global burden of diseases due to UVR have pointed out gaps in our current knowledge and understanding of UVR and warrant further research across the interdisciplinary fields to improve precision and broaden the scope of assessment for enduring results. More research is required to clarify the other beneficial effects of sun exposure and if total exposure avoidance with vitamin D supplementation from outside sources could be feasible. Further, strict sun protection measures are being followed, especially by skin types I-IV to prevent the UV–B–induced skin damage by avoiding excessive sun exposure (72). The use of sunscreens is also recommended, especially for fair-skinned individuals, but not a general rule. However, clinical dermatologists and researchers must understand the balancing effects of UV exposure and provide a shred of convincing evidence by weighing the mutagenic effect of less intense UV than its protective effects on different life forms (73, 74). This information also needs to be translated into public-friendly outcomes and could prolong many lives through positive recommendations on strict sun protection measures which require further moderation. Also, researchers need to deconstruct what it means by adequate vitamin D status when sun exposure is seriously curtailed and recommended, taking into account the associated ill effects of low sun exposure.

## Vitamin D Fortification Of Foods: A Novel Biotechnological Strategy To Curb Vitamin D Deficiency In Humans

Optimum Vitamin D in body is essential for healthy bone and organ function (75). Exposure to sunlight (ultraviolet-B) is the primary natural source of vitamin D in the skin. At the same time, only a tiny portion of the necessary amount can be acquired through diet (76). There are six factors to be considered on the optimal amount of UV exposure depending on the latitude for healthy vitamin D levels, i.e., location, time of day, outdoor weather, skin colour, total sun exposure and age. Based on these factors, every individual has their sunlight exposure requirements based on specific elements that are both intrinsic and extrinsic to the body. However, to maintain healthy blood levels of vitamin D, one should aim to get 10–30 minutes of midday sunlight several times per week, and people with darker skin may require a little more. Exposure of face and arms to the sunlight for 15–30 minutes, between 11 am–3 pm daily, should be enough to maintain adequate vitamin D status. However, geographical location remains the most important determinant of vitamin D status in the body (77). Although Vitamin D has many health benefits, it might even help lower the risk of some cancers (78). However, researchers aren’t sure what the optimal level of vitamin D is, and a lot of research is already going on to understand the intricate relationship clearly.

Further, the optimal level of UV exposure for healthy vitamin D production may increase the cancer risk. As such, the risk-benefit may vary widely due to individual susceptibility, genetic and lifestyle factors (79, 80). The fact is that it doesn’t take so much sun exposure for the body to produce required amounts of vitamin D and exposure time less than 10 to 15 minutes, two to three times a week, followed by good sun protection can make all the vitamin D on which a body can muster (81). After the required levels are achieved, the body automatically starts disposing of the excess vitamin D to avoid vitamin overload. At this point, persistent sun exposure gives nothing but sun damage without any of the presumed benefits. Research has shown that this 10-15 minute exposure to the body is enough to cause DNA damage, and every bit of it adds up throughout one’s life, producing genetic mutations that keep increasing the lifetime risk of skin cancer (82). The exact UVB wavelength that makes the body synthesize vitamin D also produces sunburn and genetic mutations that can lead to skin cancer. A study has recently found that UVA damage can start in less than a minute. This damage to the skin’s pigment cells keeps developing hours after the sun exposure ends, increasing the chances of melanoma (83). This rapid onset of DNA damage is also why researchers recommend more sun protection, not less. That is why this complex set of risks and benefits vary widely, and that guidance that addresses all of these factors is difficult to articulate.

Vitamin D deficiency has emerged as a new public health concern in recent years due to lifestyle changes and strict recommendations on avoiding excessive sun exposure. It has thus received increased attention due to its association with the increased risk of severe acute and chronic illnesses, including rickets, childhood caries, osteoporosis, infections, autoimmune diseases, cardiovascular diseases, cancer, type 2 diabetes and neurological disorders (84). On the other hand, a healthy and balanced diet is not enough to prevent vitamin D insufficiency in the body if it is not accompanied by UV exposure. However, specific approaches such as increased dietary and supplemental intakes and encouraging outdoor activities could guarantee vitamin D sufficiency. However, more recently, biotechnological processes were used to produce novel vitamin D rich or vitamin D fortified foods, which can improve vitamin D status and prevent vitamin D deficiency in high-risk individuals. Various foods were fortified during the early 20th century, including milk, other dairy products, margarine, and even beer. Initially, and since the 1940s, cow’s milk became the primary delivery vehicle for vitamin D fortification in the United States and Canada, and a carefully planned fortification policy was introduced to eliminate vitamin D deficiency and as a public health issue (85). Voluntary and mandatory fortification approaches are applied in USA and Canada, respectively. Both are entrusted to provide fortified foods with proven efficacy. Current estimates suggest that ~60% of vitamin D intake from foods in the US and Canada are attributed to fortified foods (85). Further, research has shown higher 25(OH)D serum levels in Canadians ingesting fortified milk than those not consuming it (86). In another prospective controlled trial study, 713 healthy school children aged 10-14 years were randomized to receive unfortified or milk fortified with 600 IU (15ug) and 1000 IU (25ug) of vitamin D per day for 12 weeks. The percentage of subjects having serum 25(OH)D levels >20 ng/ml (50 nmol/L) following supplementation was found 5.9%, 69.95% and 81.11% in comparison to 6.32%, 4.9% and 12%, respectively, at baseline (87), suggesting the success of the fortification policy. Some population groups do not consume fortified milk due to lactose intolerance. Prospective studies have shown that foods of plant origin such as orange juice and bread can also be used as suitable vehicles for vitamin D fortification. Although traditional fortification practices serve as an essential strategy, the introduction of novel biotechnology-based vitamin D fortification approaches will continue to attract attention. Also, fortification approaches need to be tailored to the nutritional habits of each country. In India, it is proposed that the fortification of widely consumed foods such as maida, wheat flour and rice could serve the purpose locally with fewer costs (88).

## Conclusion

UVR exposure has both positive as well as negative health effects on humans. An increase in skin cancer cases over the last 4 to 5 decades has raised various public health concerns among the scientific community and led international health organizations to develop strong sun protection measures to curb this sharp increase. However, at the same time, a growing concern about vitamin D deficiency, mostly in high-risk groups, is creating a kind of exposure dilemma. Current knowledge and understanding of the ambivalent effects of UV exposure necessitates a correct public health recommendation on optimal sun exposure based on the scientific facts and reasoning. Also, Vitamin D deficiency that has emerged as a significant public health issue can be overcome with biotechnology-based approaches like food fortification. Vitamin D fortification of foods is technically a feasible method that can address the vitamin deficiency in large population segments without modifications in lifestyle and consumption patterns. Further, biotechnology can offer viable solutions in producing new and novel vitamin D fortified foods. This can somehow lead to positive outcomes in protecting humans against the adverse effects of strict recommendations on sun protection measures.

## Author Contributions

Conceptualization and Formal analysis: SU and ST. Funding acquisition: ST. Writing - original draft: SU. Writing - review and editing: ST and SU. All authors contributed to the article and approved the submitted version.

## Conflict of Interest

The authors declare that the research was conducted in the absence of any commercial or financial relationships that could be construed as a potential conflict of interest.

## Publisher’s Note

All claims expressed in this article are solely those of the authors and do not necessarily represent those of their affiliated organizations, or those of the publisher, the editors and the reviewers. Any product that may be evaluated in this article, or claim that may be made by its manufacturer, is not guaranteed or endorsed by the publisher.
